# Postoperative alterations of sagittal cervical alignment and risk factors for cervical kyphosis in 124 Lenke 1 adolescent idiopathic scoliosis patients

**DOI:** 10.1186/s12891-021-04884-4

**Published:** 2021-11-30

**Authors:** Junyu Li, Kaige Deng, Yanchao Tang, Zexi Yang, Xiaoguang Liu, Zhongjun Liu, Feng Wei, Fengliang Wu, Hua Zhou, Yan Li, Yongqiang Wang, Weishi Li, Miao Yu

**Affiliations:** 1 Orthopedic Department, Peking University Third Hospital, 49 North Garden Road, Haidian District, Beijing, 100191 China; 2 Engineering Research Center of Bone and Joint Precision Medicine, 49 North Garden Road, Haidian District, Beijing, 100191 China; 3 Beijing Key Laboratory of Spinal Disease Research, 49 North Garden Road, Haidian District, Beijing, 100191 China; 4 Peking University Health Science Centre, 38 Xueyuan Road, Haidian District, Beijing, 100191 China

**Keywords:** Adolescent idiopathic scoliosis, pedicle screws, cervical sagittal compensation, cervical kyphosis, independent risk factors

## Abstract

**Background:**

This study aims to analyze postoperative changes of cervical sagittal curvature and to identify independent risk factors for cervical kyphosis in Lenke type 1 adolescent idiopathic scoliosis (AIS) patients.

**Methods:**

A total of 124 AIS patients who received all-pedicle-screw instrumentation were enrolled. All patients were followed up for at least 2 years. The following parameters were measured preoperatively, immediately after the operation, and at the last follow-up: pelvic incidence (PI), pelvic tilt (PT), sacral slope (SS), lumbar lordosis (LL), thoracic kyphosis (TK), global thoracic kyphosis (GTK), proximal thoracic kyphosis (PrTK), T1-slope, cervical lordosis (CL), McGregor slope (McGS), sagittal vertical axis (SVA), C2–7 SVA (cSVA), and main thoracic angle (MTA). Statistical analysis was performed to evaluate postoperative alterations of and correlations between the parameters and to identify risk factors for cervical kyphosis. Statistical significance was set at *P* < 0.05.

**Results:**

After the operation, PrTK and T1-slope significantly increased (3.01 ± 11.46, 3.8 ± 10.76, respectively), cervical lordosis improved with an insignificant increase (− 2.11 ± 13.47, *P* = 0.154), and MTA, SS, and LL decreased significantly (− 33.68 ± 15.35, − 2.98 ± 8.41, 2.82 ± 9.92, respectively). Intergroup comparison and logistic regression revealed that preoperative CK > 2.35° and immediate postoperative GTK < 27.15° were independent risk factors for final cervical kyphosis, and △T1-slope < 4.8° for a kyphotic trend.

**Conclusions:**

Postoperative restoration of thoracic kyphosis, especially proximal thoracic kyphosis, and T1-slope play a central role in cervical sagittal compensation. Preoperative CK, postoperative small GTK, and insufficient △T1-slope are all independent risk factors for cervical decompensation.

## Background

Adolescent idiopathic scoliosis (AIS) is a three-dimensional spinal deformity that affects approximately 1% ~ 4% of adolescents. Pedicle screw instrumentation is commonly used in the posterior correction for AIS patients who have a main curve over 40° [1–3], and its outcome is often considered satisfactory for deformity corrections [4–6]. For a long time, studies on AIS have typically focused on the correction of coronal imbalance. In recent years, however, surgical impacts on the sagittal curvature, especially cervical alignment, have gained increasing attention [4, 7–13], with an emerging literature that has examined postoperative cervical kyphosis (CK) [14], the correlation between cervical curvature and neck pain [15], as well as the health-related quality of life (HRQOL) [16].

Adjacent to the cervical spine, the thoracic segment has a sagittal compensatory correlation with the cervical segment [17]. Therefore, the postoperative interaction between these two segments has recently received more discussions [10–12, 18]. Hayashi et al. [8] identified preoperatively smaller cervical lordosis (CL) and smaller thoracic kyphosis (TK) as independent risk factors for postoperative CK. Zhang et al. [11] proposed that combining preoperative CL and immediate postoperative T1-slope together could predict the final CL value. The study of Zhu et al. [12] found that the thoracic inlet angle (TIA) was predictive to the postoperative CK.

According to the Lenke classification, the deformity of Lenke 1 AIS patients is located at a relatively cranial position of the main curve, which correspondingly requires a fusion surgery of more cranial segments. Some researchers believe that this specific character makes the thoracic and cervical alignment more susceptible to surgery in Lenke 1 patients. But the specific postoperative change of cervical lordosis in this Lenke type is still controversial [4, 14, 19, 20], and there is no literature to identify independent risk factors leading to cervical decompensation after operation in this group of patients.

Based on the reports mentioned above, this study aims to analyze the sagittal compensation process of the cervical spine after correctional surgeries using all-pedicle-screw instrumentation and to explore independent risk factors for postoperative cervical kyphosis in Lenke type 1 AIS patients.

## Methods

### Level of evidence

The level of evidence of this retrospective cohort study is 3.

### Data

This study retrospectively reviewed a total of 157 consecutive AIS patients who received surgeries at our institution from 2005 to 2018. Inclusion criteria were as follows: 1) diagnosed with adolescent idiopathic scoliosis; 2) classified as Lenke type 1; 3) 22 years of age or younger; 4) received posterior correction using all-pedicle-screw instrumentation; and 5) complete preoperative, postoperative and follow-up full spine radiographs at posterior-anterior and lateral positions. Exclusion criteria included: 1) diagnosed of additional scoliosis, 2) a follow-up period less than 2 years, 3) received other neuromuscular operations before, and 4) poor radiograph image quality. Following these criteria, a total of 124 patients were included. This study was approved by the institutional review board of our hospital.

All participants’ demographic data and treatment-related data were extracted through our hospital’s electronic medical record system. We measured a series of coronal and sagittal radiographic parameters and calculated their changes from the preoperative measurement to the last follow-up (Δ parameter).

### Radiographic measurements

Standing posterior-anterior and lateral radiographs were taken using the same procedure and machine. The standard posture of lateral radiography was standing upright and looking straight ahead, with knuckles placed in the supraclavicular fossa bilaterally and the arms at a 45°angle to the vertical axis. The radiographs were measured using Surgimap (Nemaris Inc., New York, NY) and we utilized the regional image enhancement function to decrease the soft tissue shadow.

The following parameters were examined: 1) Cobb angle of the main thoracic curve on the coronal plane (MTA); 2) cervical sagittal parameters, including cervical lordosis (CL), T1-slope, and McGregor slope (McGS); 3) thoracolumbar parameters, including thoracic kyphosis (TK and GTK), proximal thoracic kyphosis (PrTK), and lumbar lordosis (LL); 3) pelvic parameters, including pelvic tilt (PT), pelvic incidence (PI), and sacral slope (SS); and 4) spinal-pelvic parameters, including sagittal vertical axis (SVA) and C2–7 SVA (cSVA).

All segmental angles were measured with the Cobb method. Lordotic alignment was presented with a negative value and kyphotic were presented with a positive value. CL was measured from the lower endplate of C2 to the lower endplate of C7. PrTK, GTK, and TK were defined from the upper surface of T1, T1, and T4 to the lower surface of T4, T12, and T12, respectively. LL was measured from the upper surface of L1 to the upper surface of S1. McGregor slope and T1 slope were defined as the angle between the horizontal line and, respectively, the McGregor line and the upper endplate of T1. Pelvic measurements were conducted according to the method described by Duval-Beaupère [21]. SVA and cSVA were defined as the horizontal distance from the center point of the C7 and C2 vertebra to the posterior endpoint of the superior endplate of S1 and C7, respectively. A positive value indicated an anterior sagittal balance and a negative value indicated the opposite [22]. All work was done by two of the authors together and measurements were determined by their consensus. Disagreements were settled by consulting a third, senior author.

### Statistical analysis

The surgical outcomes were assessed with the final cervical lordosis/kyphosis as well as the changes of cervical curvature as shown on the radiographs, so as to comprehensively represent the outcome of cervical compensation. Thus, according to the cervical lordosis value at the last measurement, patients were grouped into the final cervical kyphosis group (Group K) or final cervical lordosis group (Group L); according to the change of CL value, patients were grouped into the improvement group (Group I) or deterioration group (Group D).

Statistical analysis was conducted using SPSS version 24.0 (SPSS, Chicago, IL). All measurements were expressed as mean ± standard deviation. Paired t-test was used to analyze the postoperative change of parameters, statistical significance was set at *P* < 0.05. Independent t-test and chi-square test were performed between outcome groups to screen the covariates that affected the outcomes. Variables with a *P*-value < 0.10 in intergroup comparison were included for further stepwise multivariate logistic regression using the likelihood ratio method to eliminate confounding factors. A significant factor whose 95% confidence interval of odds ratio didn’t include unity was identified as an independent risk factor. The receiver operating characteristic curve (ROC curve) was utilized to determine the best cut-off value for predicting the outcome of cervical compensation.

## Results

### Baseline data

The demographic features of the patients are shown in Table 1. The mean age was 14.2 years (range 9–22), the sex ratio was 80 females to 44 males. Median upper instrumented vertebra (UIV) was at T 3 (range T2-T4) and median fused levels were 10.5 vertebrae. The minimum follow-up period was 24 months and the mean follow-up period was 37.3 months. The mean hospital stay was 17.2 days and the mean surgery duration was 321.4 min. No intra-operative complication was reported.

**Table 1:** Demographic data of the 124 patients

ITEMS	MEAN ± SD / N / MEDIAN
**Age (years of age)**	14.2 ± 2.8
**Sex**	
**Male(N)**	44
**Female(N)**	80
**Height (cm)**	160.2 ± 12.6
**Weight (kg)**	48.1 ± 12.6
**BMI (kg/m**^**2**^**)**	18.6 ± 3.9
**Follow-up period (mon)**	37.3 ± 12.9
**VAS-lower extremities**	0 ± 0.1
**VAS-lumbar**	0.7 ± 1.46
**Oswestry-Disability-Index**	1.3 ± 3.0
**Hospital stay(d)**	17.2 ± 23.0
**Surgery duration (min)**	321.4 ± 133.3
**Total blood loss (ml)**	792.9 ± 594.2
**comorbidities**	
**≥ 1(N)**	42
**0(N)**	82
**complications**	
**≥ 1(N)**	4
**0(N)**	120
**Median upper instrumented level**	T3
**Median lower instrumented level**	L1
**Median fused levels**	10.5

### Change of sagittal parameters after surgery

All preoperative, postoperative, and the last follow-up measurements are listed in Table 2. MTA showed a significant decrease from 49.8° to 15.0°. PI, SS, TK, and GTK showed a decrease immediately postoperatively followed by a reverse change during the follow-up period, the reduction was significant at the last follow-up only in SS (− 3.0 ± 8.4) and LL (2.8 ± 9.9). By contrast, PT showed an increase immediately postoperatively followed by a decrease. Meanwhile, steady increases from preoperatively to the last follow-up were found in PrTK, T1-slope, CL, and McGS and reached significance in PrTK (3.0 ± 11.5) and T1-slope (3.8 ± 10.8).

**Table 2:** Sagittal parameters at different measurements: preoperatively, postoperatively, and at the last follow-up

	Pre-op	Post-op	Last follow-up (LFU)	***P***-value (Pre-op VS. Post-op)	***P***-value (Post-op VS. LFU)	***P***-value (Pre-op VS. LFU)	△(PRE-OP TO LFU)
**MTA(°)**	49.8 ± 18.0	15.0 ± 11.5	16.1 ± 11.4	0.000	0.301	0.000	−33.7 ± 15.4
**PI**	45.5 ± 12.8	43.4 ± 13.9	44.2 ± 13.2	0.176	0.718	0.140	−3.4 ± 11.7
**PT**	6.9 ± 12.6	8.7 ± 10.3	6.8 ± 10.1	0.217	0.067	0.669	−0.4 ± 9.6
**SS**	39.8 ± 9.1	34.7 ± 8.8	37.5 ± 8.4	0.000	0.015	0.010	−3.0 ± 8.4
**LL**	− 56.1 ± 12.0	−44.3 ± 12.5	−53.2 ± 11.1	0.000	0.000	0.029	2.8 ± 9.9
**TK**	30.4 ± 18.0	25.2 ± 11.8	28.8 ± 12.4	0.000	0.000	0.313	−1.6 ± 12.4
**GTK**	33.6 ± 17.6	28.6 ± 11.7	35.6 ± 12.6	0.029	0.000	0.138	2.4 ± 12.2
**PrTK**	7.0 ± 9.1	8.1 ± 9.9	10.0 ± 10.1	0.309	0.180	0.041	3.0 ± 11.5
**SVA**	−18.8 ± 39.9	12.3 ± 33.9	−15.1 ± 29.2	0.000	0.000	0.495	3.8 ± 42.3
**cSVA**	21.4 ± 10.4	23.4 ± 10.7	21.2 ± 11.4	0.082	0.169	0.876	−0.2 ± 10.5
**T1-SLOPE**	16.7 ± 12.3	18.8 ± 8.0	19.7 ± 9.0	0.120	0.425	0.022	3.8 ± 10.8
**CL**	−3.1 ± 16.4	−4.0 ± 12.1	−5.1 ± 14.6	0.521	0.573	0.154	−2.1 ± 13.5
**McGS**	3.2 ± 8.6	5.2 ± 7.3	5.4 ± 21.6	0.045	0.977	0.502	2.4 ± 22.1

### Risk factors for final cervical kyphosis

Intergroup comparison was performed regarding all radiographic variables. Results of this univariate analysis were shown in Table 3 and Table 4.

**Table 3:** Univariate analysis between Group K and Group L

Intergroup Comparison measurement	GROUP K vs. GROUP L			
	Pre-op	Post-op	Last follow-up	△parameter
**MTA**	49.8 ± 16.2 vs. 49.7 ± 19.3	13.0 ± 11.8 vs. 16.2 ± 11.3	16.2 ± 11.2 vs. 16.0 ± 11.6	−33.7 ± 13.2 vs. -33.7 ± 16.8
**PI**	47.8 ± 14.4 vs. 44.0 ± 11.6	47.7 ± 15.3 vs. 40.7 ± 12.3*	48.3 ± 13.6 vs. 41.4 ± 12.3**	0.5 ± 9.2 vs. -5.9 ± 12.5**
**PT**	11.1 ± 16.2 vs. 4.3 ± 9.0**	10.3 ± 11.4 vs. 7.7 ± 10.0	10.8 ± 10.2 vs. 4.1 ± 9.3**	−0.3 ± 13.0 vs. -0.5 ± 6.8
**SS**	40.0 ± 9.2 vs. 39.7 ± 9.2	37.4 ± 9.2 vs. 33.1 ± 8.2*	37.6 ± 8.1 vs. 37.4 ± 8.8	−2.5 ± 7.1 vs. -3.3 ± 9.2
**LL**	−52.6 ± 11.7 vs. -58.2 ± 11.9*	− 43.3 ± 12.5 vs. -44.9 ± 12.6	− 51.8 ± 11.1 vs. -54.2 ± 11.1	0.9 ± 9.8 vs. 4.1 ± 9.9
**TK**	18.9 ± 12.9 vs. 37.8 ± 170.***	18.2 ± 6.9 vs. 29.7 ± 12.1***	22.5 ± 9.8 vs. 32.8 ± 12.2***	3.6 ± 12.2 vs. -4.9 ± 11.4***
**PrTK**	5.1 ± 6.3 vs. 8.2 ± 10.4	8.1 ± 8.3 vs. 8.1 ± 10.9	7.7 ± 7.1 vs. 11.5 ± 11.6	2.8 ± 6.6 vs. 3.2 ± 13.8
**GTK**	23.0 ± 12.2 vs. 39.9 ± 17.3***	20.7 ± 8.9 vs. 33.4 ± 10.7***	26.9 ± 9.8 vs. 41.2 ± 10.9***	4.3 ± 11.7 vs. 1.2 ± 12.5
**SVA**	−23.7 ± 42.1 vs. -15.7 ± 38.6	11.7 ± 28.2 vs. 12.7 ± 37.4	−19.0 ± 29.4 vs. -12.5 ± 29.2	4.7 ± 37.0 vs. 3.2 ± 45.9
**CSVA**	23.5 ± 12.1 vs. 20.0 ± 9.2	25.6 ± 9.7 vs. 22.0 ± 11.1	23.1 ± 10.2 vs. 20.0 ± 12.1	−0.4 ± 7.4 vs. -0.1 ± 12.1
**T1-slope**	9.2 ± 9.0 vs. 21.2 ± 11.9***	14.5 ± 6.5 vs. 21.5 ± 7.8***	12.7 ± 6.4 vs. 24.1 ± 7.4***	4.3 ± 9.1 vs. 3.5 ± 11.8
**CL**	10.5 ± 12.9 vs. -11.1 ± 12.5***	3.7 ± 10.1 vs. -8.9 ± 10.7***	9.1 ± 7.6 vs. -14.0 ± 10.3***	−0.5 ± 13.9 vs. -3.1 ± 13.3
**McGS**	4.2 ± 6.9 vs. 2.6 ± 9.6	7.0 ± 5.5 vs. 4.2 ± 8.1	11.3 ± 33.1 vs. 1.7 ± 7.4*	7.5 ± 33.7 vs. -0.8 ± 8.5

Method: independent t-test, chi-square test. *P < 0.10, **P < 0.05, ***P < 0.01

**Table 4:** Univariate analysis between Group D and Group I

Intergroup Comparison	GROUP D vs. GROUP I			
measurement	Pre-op	Post-op	Last follow-up	△parameter
**MTA**	51.7 ± 21.5 vs. 48.5 ± 15.3	15.6 ± 14.9 vs. 14.5 ± 8.7	17.4 ± 13.3 vs. 15.2 ± 10.0	−34.3 ± 15.3 vs. -33.3 ± 15.6
**PI**	47.0 ± 12.2 vs. 44.5 ± 13.2	44.6 ± 11.5 vs. 42.5 ± 15.4	44.1 ± 10.6 vs. 44.3 ± 15.0	−2.9 ± 6.8 vs. -3.8 ± 14.2
**PT**	6.1 ± 8.1 vs. 7.5 ± 14.9	7.9 ± 7.8 vs. 9.3 ± 11.8	6.2 ± 6.0 vs. 7.3 ± 12.4	0.1 ± 6.0 vs. -0.8 ± 11.5
**SS**	40.9 ± 10.6 vs. 39.1 ± 8.0	36.7 ± 9.1 vs. 33.3 ± 8.4	38.0 ± 9.0 vs. 37.1 ± 8.2	−3.0 ± 7.7 vs. -3.0 ± 9.0
**LL**	−57.0 ± 11.7 vs. -55.5 ± 12.4	−45.3 ± 14.6 vs. -43.6 ± 11.0	−53.6 ± 11.6 vs. -53.0 ± 10.8	3.4 ± 11.0 vs. 2.5 ± 9.3
**TK**	31.3 ± 20.5 vs. 29.8 ± 16.3	24.4 ± 13.5 vs. 25.8 ± 10.6	26.3 ± 12.1 vs. 30.5 ± 12.4	−5.0 ± 13.4 vs. 0.7 ± 11.2*
**PrTK**	7.2 ± 9.1 vs. 6.9 ± 9.2	5.1 ± 11.4 vs. 10.2 ± 8.3**	9.8 ± 8.5 vs. 10.1 ± 11.3	2.9 ± 8.8 vs. 3.1 ± 13.0
**GTK**	39.0 ± 17.6 vs. 30.1 ± 16.9*	26.4 ± 11.1 vs. 30.1 ± 12.1	33.2 ± 11.4 vs. 37.2 ± 13.2	−4.9 ± 11.5 vs. 7.0 ± 10.3***
**SVA**	−18.6 ± 35.6 vs. -19.0 ± 42.8	17.4 ± 26.0 vs. 9.0 ± 38.1	−17.0 ± 29.7 vs. -13.8 ± 29.2	1.6 ± 39.0 vs. 5.2 ± 44.7
**CSVA**	22.9 ± 11.7 vs. 20.4 ± 9.6	22.8 ± 11.2 vs. 23.8 ± 10.4	22.6 ± 10.7 vs. 20.2 ± 11.9	−0.3 ± 8.5 vs. -0.2 ± 11.6
**T1-slope**	21.5 ± 12.5 vs. 13.6 ± 11.3**	17.8 ± 7.6 vs. 19.5 ± 8.4	17. 6 ± 8.0 vs. 21.1 ± 9.4	−2.3 ± 11.2 vs. 8.0 ± 8.4***
**CL**	−12.0 ± 14.3 vs. 2.6 ± 15.2***	−6.4 ± 13.4 vs. -2.5 ± 11.1	0.0 ± 14.2 vs. -8.5 ± 14.1**	11.0 ± 8.2 vs. -11.0 ± 7.8***
**McGS**	2.4 ± 9.8 vs. 3.6 ± 7.9	5.9 ± 8.8 vs. 4.8 ± 6.5	10.3 ± 32.9 vs. 2.2 ± 6.7	8.1 ± 33.3 vs. -1.4 ± 7.2

Method: independent t-test, chi-square test. *P < 0.10, **P < 0.05, ***P < 0.01

There were 48 patients in Group K and 76 in Group L. With an exception of the final CL value, intergroup univariate analysis showed that all the following parameters were statistically different between groups and were thus regarded as potential risk factors: preoperative PT, LL, TK, GTK, T1-slope, and CL; immediate postoperative PI, SS, TK, GTK, T1-slope, and CL; PI, PT, TK, GTK, T1-slope, and McGS at the last follow-up; and △PI and △TK (Table 3).

There were 50 patients in Group D and 74 in Group I. The potential risk factors identified through intergroup comparison included: preoperative GTK and T1-slope; immediate postoperative PrTK; CL at the last follow-up; and △TK, △GTK, and △T1-slope (Table 4).

Since this study aimed to identify factors affecting the ultimate outcome of cervical alignment, only preoperative, postoperative, and △parameters were useful variables for our purposes. In our multivariate logistic regression model, we included these potential risk factors as covariates while treating final cervical kyphosis or cervical alignment deterioration as dependent factors. As the model revealed, preoperatively larger cervical kyphosis or smaller cervical lordosis (*P* = 0.008, OR = 1.267, 95%CI: 1.064 ~ 1.515), as well as postoperatively smaller GTK (*P* = 0.006, OR = 0.806, 95%CI: 0.692 ~ 0.940), were independent factors for final cervical kyphosis, while smaller △T1-slope was an independent risk factor for a kyphotic trend of the cervical spine (*P* = 0.002, OR = 0.766, 95%CI: 0.647 ~ 0.907). Some of the lumbar, pelvic and global spinal parameters also had an impact on the final cervical alignment in single variate analysis, however, were eliminated as subsidiary risk factors by the stepwise multivariate logistic regression.

**Fig. 1:**
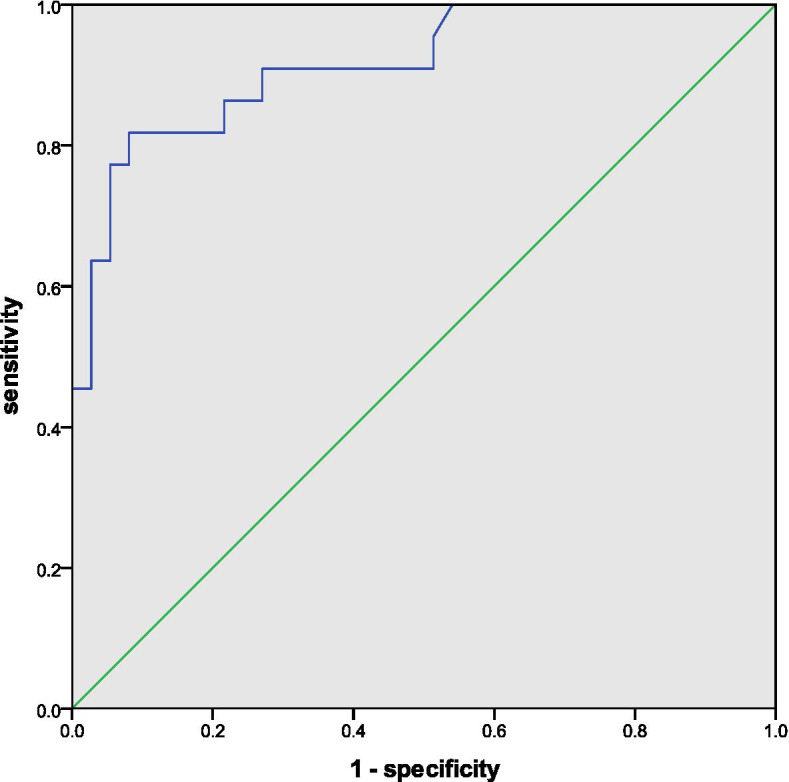
ROC curve displaying the predictive power of preoperative CL for final cervical kyphosis

**Fig. 2:**
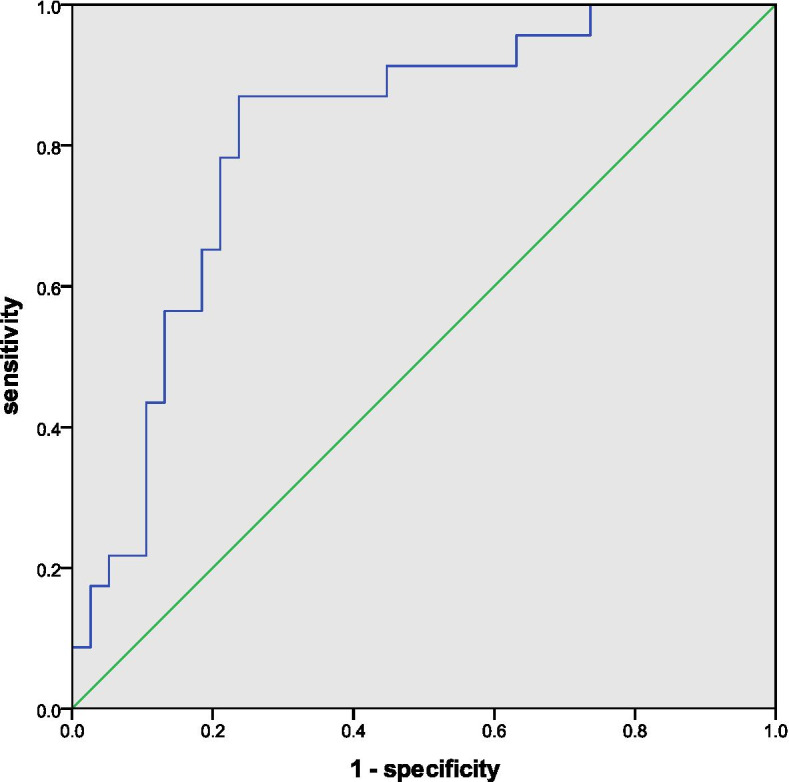
ROC curve displaying the predictive power of postoperative GTK for final cervical kyphosis

**Fig. 3:**
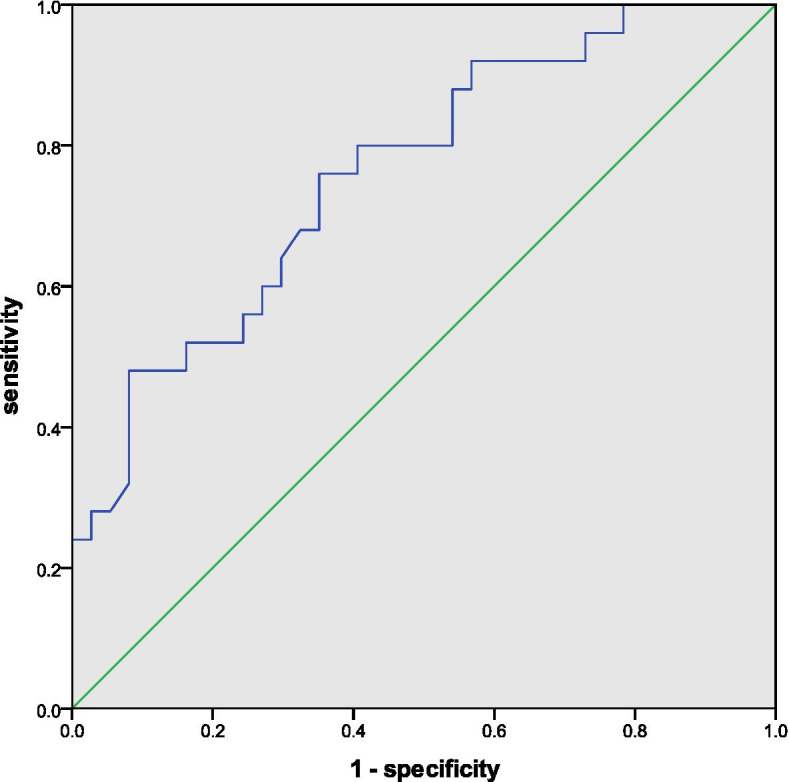
ROC curve displaying the predictive power of △T1-slope for a kyphotic trend of cervical alignment

We applied the ROC curve analysis to these three risk factors, which proved that both preoperative CL and postoperative GTK were good predictive parameters for final cervical kyphosis, with an area under the curve (AUC) of 0.912 for the former and an AUC of 0.811 for the latter. As for the acceptable predictive power of △T1-slope for the kyphotic trend, the AUC was 0.761 (Figs. 1, 2 and 3). ROC curve analysis also determined that preoperative CL > 2.35°, postoperative GTK < 27.15°, and △T1-slope < 4.80°, were the preferred cut-off value for risk stratification with the best sensitivity and specificity.

## Discussion

### Postoperative change of radiographic parameters

The results of this study indicated a satisfactory surgical outcome, with a correction rate of 67.70% at the last follow-up, which is consistent with former reports [4]. Some scholars believed that on the sagittal plane, cervical alignment was correlated to thoracic alignment as a compensation mechanism to keep a horizontal vision [17]. Hence, we must understand the surgical impacts on the thoracic spine prior to analyzing the postoperative compensation of the cervical spine.

So far, the postoperative change of thoracic kyphosis in Lenke 1 patients is still controversial. Most studies reported decreased TK after all-pedicle-screw instrumentation. Legarreta et al. [4] observed that curve correction significantly reduced TK in preoperative normal-kyphotic (20° ~ 40°) and hyper-kyphotic groups (> 40°). Likewise, Hwang et al. [14] proposed that surgery with all pedicle screws constructs could flatten the thoracic sagittal curvature in Lenke types 1 and 2 patients. In our research, both TK and GTK significantly decreased in the early postoperative period, which was in line with previous studies. AIS is a three-dimensional spinal deformity, and more correction on the coronal plane in surgeries with all pedicle screws may account for the reduction of thoracic kyphosis on the sagittal plane [23]. Additionally, there were other researches suggesting that TK could remain unchanged or even increase after correctional surgeries [9, 19, 20]. The variance in outcomes might be accounted for by the mixed Lenke types or different surgical techniques in these studies.

Nonetheless, loss of thoracic kyphosis did not necessarily lead to cervical decompensation. Our results suggested that PrTK and T1-slope both had a remarkable increase by 3.0° and 3.8°, although TK decreased postoperatively. The cervical spine was subsequently subject to an increased lordosis. Similarly, we not only observed corresponding changes in PrTK, T1-slope, and CL, but also found a significant correlation between PrTK and T1-slope immediately after the operation. In previous studies, Charles et al. [19] observed a significant improvement in PrTK and T1-slope, along with an associated improvement in CL. Ketenci et al. [9] found that the change of CL was consistent with that of PrTK. They proposed that PrTK could affect T1-slope, thereby triggering changes in CL. We considered our results of the change of PrTK, T1-slope, and CL reliable. On the one hand, the consistency among the changes of these three parameters echoed with former researches [9, 19] as well as the results of our correlation analysis, on the other hand, despite the small amount of change in these parameters, the standard posture, the two-men measurement procedure and a large cohort with over 120 patients could assure the significance of results. The small enlargement of cervical lordosis (2.1°) could be explained by the relatively close preoperative CL value to asymptomatic teenagers (− 3.09° vs. − 4.8° [24]).

Throughout the whole study period, PI, PT, SS, LL, TK, GTK, SVA, and cSVA all initially decreased (or increased) and then increased (or decreased). The synchronous changes of these parameters reflected the cone of the economy in the maintaining of sagittal balance described by Pepke et al. [25]. Interestingly, a positive correlation between T1-slope and SVA was also observed in all three measurements, which was in line with the conclusion of Knott et al. [26] and indicated that T1-slope was a great indicator for sagittal balance. Furthermore, despite the temporal anterior displacement of SVA after surgery, the upper thoracic-cervical curvature showed steady improvement in the immediate and long-term postoperative period, further proving the reliability of our results.

These results suggested that an unfused upper thoracic segment and the cervical-thoracic junction played a more vital role in the improvement of CL, compared to the global or instrumented thoracic segment.

### Independent factors for kyphosis of cervical spine

We identified the risk factors for cervical kyphosis, which could facilitate the prevention of cervical sagittal decompensation. Compared to the patients in Group L, those in Group K showed smaller preoperative TK, GTK, and T1-slope. This result was in line with previous studies, suggesting that the degree of preoperative TK was significantly related to the occurrence of postoperative CK [7]. Specifically, patients with larger TK tend to have better restoration of CL [15]. Hayashi et al. [8] noted that patients with final hyper-CK had significantly smaller TK and larger CK preoperatively. We found that immediate postoperative GTK < 27.15° was a risk factor for final CK and that in the early postoperative period, segmental instrumentation had a flattening effect on thoracic kyphosis. Although TK was generally restored at the last follow-up, we need to pay extra attention to the correction of thoracic curvature during the surgery to prevent the occurrence of small GTK.

Through logistic regression, we confirmed that preoperative CK > 2.35° was an independent risk factor for final CK. Studies have shown that the preoperative value of CL can predict the final value of CL [11] and that smaller preoperative CL may increase the risk of final CK [8]. These findings, along with our results, suggest that patients with larger CK should be cautiously corrected due to the risk of postoperative cervical decompensation.

Intergroup comparison between Group I and Group D finally identified △T1-slope<4.8° as an independent risk factor for a kyphotic trend of the cervical spine. Previously, Cho et al. [7] observed a significant difference in T1-slope between patients with postoperative CSA restoration and those with CSA aggravation. Therefore, our study confirmed that cervical alignment and its postoperative change were significantly affected by T1-slope, suggesting that the surgeon should pay attention to the restoration of T1-slope to facilitate that of a normal cervical curvature.

### Limitations

There are a few limitations of this study. Firstly, our study largely relied on the radiographs of the patients and we had no access to the clinical outcomes. We assume that the improvement of cervical lordosis showed in this study could lead to a lower incidence of neck pain and other poor life-quality-related outcomes. Secondly, the two-year follow-up period was relatively short. Last, due to the lack of bending radiography in some patients, we didn’t access the flexibility of the instrumented spine. To verify our conclusions, a prospectively designed cohort study with long-term follow-up, assessment of the flexibility of the spine, and clinical outcomes (VA, ODI, SF-36, JOA scoring, etc.) is needed. Regardless, this study was among the first attempts to identify independent risk factors affecting postoperative cervical alignment and to analyze the sagittal compensatory mechanism of the cervical spine in Lenke 1 AIS patients who received surgical corrections using all pedicle screws. Our results may contribute to surgical planning and prognosis predicting.

## Conclusions

Postoperative restoration of thoracic kyphosis, especially proximal thoracic kyphosis, and T1-slope play a central role in cervical sagittal compensation. Preoperative CK > 2.35°, postoperative GTK < 27.15°, and △T1-slope<4.80° are all independent risk factors for cervical decompensation.

## Data Availability

The datasets generated and analyzed during the current study are not publicly available due privacy concerns but are available from the corresponding author on reasonable request and with permission of Peking University Third Hospital.

## Abbreviations

AIS: Adolescent idiopathic scoliosis
CK: Cervical kyphosis
CL: Cervical lordosis
TK: Thoracic kyphosis
MTA: Main thoracic angle
McGS: McGregor slope
GTK: Global thoracic kyphosis
PrTK: Proximal thoracic kyphosis
LL: Lumbar lordosis
PT: Pelvic tilt
PI: Pelvic incidence
SS: Sacral slope
SVA: Sagittal vertical axis
cSVA: Cervical sagittal vertical axis
UIV: Upper instrumented vertebra
LIV: Lower instrumented vertebra
VAS: Visual Analogue Scale
BMI: Body mass index
ROC: Receiver operating characteristic
